# Clinical characteristics of schizophrenia, depression, and Alzheimer’s diseases among older adults: a retrospective study of 271 consecutive admissions

**DOI:** 10.3389/fpsyt.2025.1486626

**Published:** 2025-03-12

**Authors:** Wen Wang, Junrong Ye, Yanheng Wei, Jiawei Huang, Haoyun Wang, Fei Liu, Shengwei Wu, Jialan Wu, Zezhi Li, Jianxiong Guo, Aixiang Xiao

**Affiliations:** ^1^ Geriatric Neuroscience Center, The Affiliated Brain Hospital, Guangzhou Medical University, Guangzhou, China; ^2^ Key Laboratory of Neurogenetics and Channelopathies of Guangdong Province and the Ministry of Education of China, Guangzhou Medical University, Guangzhou, China; ^3^ School of Nursing, Guangzhou Medical University, Guangzhou, China; ^4^ Department of Nursing, The Affiliated Brain Hospital, Guangzhou Medical University, Guangzhou, China; ^5^ Affiliated Cancer Hospital and Institute of Guangzhou Medical University, Guangzhou, China; ^6^ Kiang Wu Nursing College of Macau, Macao, Macao SAR, China; ^7^ Department of Traditional Chinese Medicine, The Affiliated Brain Hospital, Guangzhou Medical University, Guangzhou, China; ^8^ Department of Geriatric Psychiatry, The Affiliated Brain Hospital, Guangzhou Medical University, Guangzhou, China; ^9^ Adult Psychiatry Department, The Affiliated Brain Hospital, Guangzhou Medical University, Guangzhou, China

**Keywords:** aged, Alzheimer’s diseases, depression, schizophrenia, characteristics

## Abstract

**Objective:**

This study aims to identify the clinical characteristics of schizophrenia, depression, and AD among older adults.

**Methods:**

General information of patients was collected, including diagnosis, age, gender, level of education, marital status, drinking behavior, smoking behavior, course of mental disorder, type of admission, history of modified electroconvulsive therapy (MECT) and hospitalization period. The Brief Psychiatric Rating Scale (BPRS), Geriatric Depression Scale (GDS), Generalized Anxiety Disorder 7-Item Scale (GAD-7), Insight and Treatment Attitudes Questionnaire (ITAQ), and Mini-Mental State Examination (MMSE) were employed to evaluate the participants’ mental status. The Functional Activities Questionnaire (FAQ), Social Support Rating Scale (SSRS), Barthel ADL Index, Standardized Swallowing Assessment (SSA), and Mini-Nutritional Assessment (MNA) were applied to measure social and daily living function. The Nurses’ Global Assessment of Suicide Risk (NGASR) and The Brøset Violence Checklist (BVC) were used to assess the patients’ risk of suicide.

**Results:**

Totally 271 participants were recruited, the numbers of participants with schizophrenia, depression, and Alzheimer’s diseases (AD), were 81 (29.9%), 85 (31.4%), and 105 (38.7%), respectively. One-way ANOVA was used to compare the variance of the crude score results among three groups of subjects. The results showed that patients with depression had the highest GDS total score, followed by patients with AD, and patients with schizophrenia had the lowest score (*P* < 0.001). The total scores of GAD-7 and ITAQ in patients with depression were higher than those in patients with AD and schizophrenia (*P* < 0.001). The total score of MMSE in patients with schizophrenia and depression was higher than that in patients with AD (*P* < 0.001). The incidence of circulatory system diseases in patients with depression and AD was higher than that in patients with schizophrenia (*P* < 0.05). The incidence of respiratory system diseases in patients with AD was highest, followed by patients with schizophrenia, and patients with depression had the lowest incidence (*P* < 0.05). The incidence of nervous system diseases in patients with AD was highest, followed by patients with depression, and patients with schizophrenia had the lowest incidence (*P* < 0.05). The total scores of FAQ and SSA in patients with AD were higher than those in patients with schizophrenia and depression (*P* < 0.001), while patients with depression had statistically lower SSRS scores than patients with schizophrenia and patients with AD (*P* < 0.05). Furthermore, patients with AD had lower Barthel ADL Index scores and water-swallowing test (*P* < 0.001). MNA scores of patients with schizophrenia were higher than those of patients with depression and AD, with statistical significance (*P* < 0.05). The NGASR scores of patients with depression were higher than those of patients with schizophrenia and AD, which was statistically significant (*P* < 0.001). Patients with AD had the highest BVC total score, followed by that of patients with schizophrenia and patients with depression had lowest score, and the difference was statistically significant (*P* < 0.05).

**Conclusions:**

Patients with geriatric psychosis may experience abnormalities in various aspects that influenced daily living, including disorders of thinking, cognition, emotion, and behavior. Patients with schizophrenia have cognitive impairment. Cognitive training and medication are important. Patients with depression were considered to be at a greater risk for suicide compared to those with schizophrenia and AD. Active clinical measures must be adopted to improve patients’ depressive symptoms, change their suicidal attitudes, and enhance their self-confidence. Patients with AD were prone to respiratory and neurological diseases. Treatment of respiratory infections and hypoxia and other respiratory diseases would be necessary, and cognitive function training should be conducted. In addition, regarding to high risk of swallowing disorders and malnutrition, swallowing function training should be carried out to ensure food intake and prevent malnutrition. Driven by psychiatric symptoms, violent behavior was prevalent, thus effective communication and de-escalation techniques are needed. Although the symptoms of these three diseases are different, timely professional intervention and support from family members are urgently needed.

## 1 Introduction

Population aging has become a major challenge worldwide. A significant shift in the global age structure can be ascribed to a decline in the birth rate and increase in the life expectancy in recent years (1). In 2010, the United Nations Population Program predicted that the proportion of people aged above 80 years old will increase fourfold by 2050 (2). Zhang et al. (2021) estimated that the number of people aged over 65 years old would increase to 19.1% of the world’s population (3). Although aging population had become a global threat, empirical findings had revealed that aging population would have greater impacts on low and middle income countries (LMICs) than developed countries in the near future.

By 2050, the global population aged above 60 years is projected to reach 2 billion, and approximately 80% of them would be from LMICs (4). China, one of the largest LMICs with great amount of aged population, is witnessing a rapid growth in the population of older adults. In 2020, residents aged over 65 years old accounted for approximately 13.5% of the national population, indicating that the country had entered the “aging society.” From 1980 to 2020, the elderly population in China had been continuously increasing. During the period, the proportion of adults aged 60 and above increased sharply, from 6.9% to 18.7%. By 2020, the elderly population size was 264.0 million (5). Cho et al. (2020) estimated the number of senior citizens in China, and their results suggested that the percentage of citizens aged above 80 years would annually increase until 2050 (6). As the second most populous country worldwide, major demographic changes had brought great challenges to health care service of the elderly in China (7–9). As a result of aging population, unexpected influential social issues had occurred. The number of older adults facing chronic diseases, disability, and care dependency had increased significantly, and concerns about older adults’ mental health had risen (10). As symptomatic treatments solely focus on physical recovery, the mental illness caused by chronic diseases and disability had been ignored to some extent.

Empirical studies demonstrated that older adults had a huge demand of mental health care (11). Notably, more than one out of five adults aged above 60 years were reported to be diagnosed with at least one mental disorders (12). In addition to impacting the quality of life of elderly individuals, mental disorders are likely to exacerbate physical illnesses, leading to higher morbidity and mortality rates (13). An empirical study found that mental disorders in elderly people were frequently accompanied by cognitive disorders, such as perceptual disorders and memory disorders (14). Furthermore, the patients’ physical functions began to deteriorate, and many patients were surrounded by varying degrees and types of physical illness. Consequently, a number of these mental illnesses were chronic, which would need a lengthy hospital stay with a high recurrence rate, and most patients were hospitalized repeatedly (15). This situation had not only significantly increased the requirement for medical assistance and care for elderly people with mental illness but had also managed to make caregiving complicated and burdensome. Elderly individuals would physically and mentally benefit from appropriate nursing care. A research in 2021 showed that, the proportion of hospitalized elderly patients with mental disorders accounted for 20.2% of total hospitalized psychiatric patients. Among 7086 hospitalized elderly patients with mental disorders, schizophrenia and depression accounted for 14.4% and 16.7%, respectively (16). Previous researches had examined the clinical characteristics of patients with schizophrenia, depression and Alzheimer’s diseases (AD) (17). Hou et al. (2017) reported that schizophrenic patients generally had sleep problems and poor quality of life. A meta-analysis by Xu et al. (2022) indicated that impairments in facial emotion recognition was a common clinical characteristic observed in individuals with schizophrenia, such facial emotion recognition difficulties predicted subsequent declines in work performance, social participation, and independent living skills among older patients with schizophrenia (18). Besides, a multi-center cross-sectional study of patients who were 65 years old or older of 13 primary care clinics in Wuhan showed that certain demographic variables, psychosocial factors, and major medical conditions were significantly associated with the severity of depressive disorders of older patients (19). In addition, Zhong et al. (2020) found that patients with unipolar disorder (UD) depression had less impairment in cognitive function among all domains than those with bipolar disorder (BD) depression, and had shorter P300 latency than BD depression patients (20). Understanding the clinical characteristics of major mental disorders could help in developing effective interventions as well as optimizing the allocation of medical resources. To this end, we analyzed 271 patients consecutively admitted to a public provincial psychiatric hospital and presented the clinical characteristics of mental disorders.

This study aimed to determine the clinical characteristics of admitted aged patients with mental disorders (schizophrenia, depression and AD), and to shed lights on developing targeted effective nursing interventions.

## 2 Methods

### 2.1 Study design and participants

A consecutive sampling method was used to recruit 271 hospitalized patients with schizophrenia, depression and AD in the Affiliated Brain Hospital, Guangzhou Medical University from February 2021 to March 2022. All participants or their family member are willing to sign the written informed consent. Inclusion criteria: (a) in accordance with International Classification of Diseases-10 (ICD-10) diagnostic criteria for schizophrenia, depression and AD; (b) age 60 years and above; (c) no sedatives or other medications that affect blood pressure and heart rate have been taken in the last two weeks; (d) be able to respond naturally and complete scale rating; (e) willingness to sign the written informed consent. Exclusion criteria: (a) poor compliance and inability to cooperate with questionnaire completion; (b) those with severe mental retardation, hearing disability; (c) combined severe physical diseases, such as cardiovascular, hepatic, or renal diseases.

### 2.2 Instruments

#### 2.2.1 General information questionnaire

General information questionnaire was designed to collect age, gender, education, marital status, alcohol consumption, smoking, course of mental disorder, type of admission, history of modified electroconvulsive therapy and hospitalization period.

#### 2.2.2 Psychiatric status

The Brief Psychiatric Rating Scale (BPRS) contains 18 items with five types of factors that are rated on a scale of 1 to 7, including the anxiety–depression factor, lack of vitality factor, thought disorder factor, activation factor, and hostile suspicion factor (21). BPRS is a well-established instrument extensively utilized to assess the severity of positive, negative, general and affective symptoms in individuals with severe mental disorders, particularly those diagnosed with schizophrenia (22). In this study, Cronbach’ s α of the scale was 0.733.

The Geriatric Depression Scale (GDS) was introduced by Brink and Yesavage in 1982. GDS was specifically developed for the evaluation of depressive symptoms in older adults (23). The scale has 30 items, including depressed mood, reduced activity, agitation, and self-reported pain, among others. Each item is followed by a number of items in parenthesis indicating the degree of depression. A score of 1 is given to each question if it is consistent with one of the items, with 1–10 being normal, 11–20 being mild depression, and 20 or more being moderate depression (23, 24). In this study, Cronbach’ s α of GDS was 0.948.

The Generalized Anxiety Disorder 7-Item Scale (GAD) is a screening tool to evaluate the severity of symptoms of generalized anxiety disorder that consists of items that are rated on a score ranging from 0 to 3, from “never” to “almost every day.”. The GAD-7 score of ≥ 10 was used as the cut-off value for screening for generalized anxiety disorder. The cut-off marks for mild, moderate, and severe generalized anxiety disorder were 5, 10, and 15, respectively (25). GAD had been widely adopted in various studies as a screening tool for anxiety symptoms among adults (26). In this study, Cronbach’ s α was 0.860.

The Insight and Treatment Attitude Questionnaire (ITAQ), developed by McEvoy (1989), defines self-awareness as “recognizing that one has a disease and requesting treatment,” and its main purpose is to assess schizophrenic patients’ awareness of their illness and their attitudes toward taking medication. The questionnaire contains 11 questions and is scored on a scale of 0 to 2: 0 for “no self-knowledge,” 1 for “partial self-knowledge,” and 2 for “full self-knowledge.” The questionnaire has the lowest score of 0 and a highest score of 22. The higher the score, the greater the patient’s self-awareness (27). In the study, Cronbach’ s α was 0.941.

The Mini-Mental State Examination (MMSE) was developed by Folstein in 1975. MMSE is one of the most extensively utilized tools for assessing cognitive function in older adults and predicting the onset of AD (28). The MMSE has 19 items. Items 1–5 are time-oriented, while items 6–10 are place-oriented. Item 11 is divided into three sub-items for verbal immediate memory, whereas item 12 is divided into five sub-items that examine attention and computation. Item 13 is divided into three sub-items to check short term memory. Item 14 is broken down into two sub-items for naming objects. The verbal retelling is item 15, and item 16 is concerned with reading comprehension. Item 17 assesses verbal comprehension, and item 18 assesses verbal communication. Finally item 19 is concerned with graphic tracing. The MMSE has a total score range of 0–30 (29). In this study, Cronbach’ s α was 0.799.

#### 2.2.3 Social and daily life functions

The Functional Activities Questionnaire (FAQ) is a commonly used scale for evaluating patients with early AD. FAQ is a screening tool for social dysfunction and adopted in studies of older adults. The scale consists of 10 questions, including: (1) using communication tools; (2) organizing objects; (3) shopping by oneself; (4) skillful activities; (5) using electrical appliances; (6) preparing meals; (7) learning about new things; (8) paying attention and understanding; (9) remembering appointments; and (10) going out alone. The rating index is the sum of the items’ scores on a 4-point scale ranging from 0 to 3. The higher the score, the greater the severity of the impairment (30). In the study, Cronbach’ s α of FAQ was 0.986.

The Social Support Rating Scale (SSRS) developed by Xiao Shuiyuan (1994) is widely used to assess levels of social support and adopted in various studies among different patients in China (31, 32). This scale includes three dimensions: subjective support, objective support, and social support utilization. For items 1–4 and 8–10, only one item was selected for each item, and 1, 2, 3, and 4 points were assigned to items 1, 2, 3, and 4, respectively. Item 5 was divided into four items, A, B, C, and D, and the total score was given for each item ranging from none to full support, and 1–4 points were given for items 6 and 7, respectively. If the respondents answered “the following sources,” they were given several points for each source. The overall score is the sum of 10 items: 1, 3, 4, and 5 are considered subjective support, 2, 6, and 7 are considered objective support, and 8, 9, and 10 are considered utilization of support (33). In the study, Cronbach’ s α of SSRS was 0.766.

The Activities of Daily Living (ADL) scale was developed by Lawton and Brody in 1969 and consists of the Barthel Index, a 10-item scale with four functional levels as per the patient’s need for help and the degree of that need. The higher the score, the more independent and the less dependent the patient is. The assessment was performed by asking the elderly about each item and could also be based on report. ADL was mainly used to assess the ability of the subject in daily life (34). Impairments in activities of daily living are common in older adults with mental disorders, such as AD, depression, and schizophrenia, and can significantly impact their independence and quality of life. The scale is suitable for assessing older adults with mental disorders (35). In this study, Cronbach’ s α was 0.929.

The water-swallowing test, or the Kubota drinking test, was first published in Japan in 1982. In this test, the patient is instructed to drink 30 ml of warm water while sitting up straight, and the amount of time needed as well as any choking symptoms are noted. (First degree: able to swallow 30 ml water without coughing or choking in one sitting; second degree: swallowed 30 ml water in twice or more times without choking or coughing; third degree: swallowed 30 ml water for once, but with choking; fourth degree: able to swallow 30 ml water in small portions but with choking; fifth degree: frequent choking and coughing, unable to swallow 30 ml water). Research had demonstrated a significantly high prevalence of dysphagia among patients with mental disorders, particularly those with cognitive impairment and severe AD (36). This test could accurately assess the patient’s swallowing function and ascertain the degree of swallowing dysfunction (37). In the study, Cronbach’ s α was 0.895.

The Standard Swallowing Assessment (SSA) scale was designed and proposed by Ellul et al. in 1996 as a method applied to assess swallowing function. From easy to difficult, the three components of this scale are: clinical assessment, which rates a person’s level of consciousness, control over their head and body, normal breathing pattern, normal lip closure, abnormal soft palate movement, abnormal laryngeal function, pharyngeal reflex, and spontaneous cough; swallowing five milliliters of water three times and observing whether water flows; and effective laryngeal movement. The third phase can be continued if no abnormalities are detected in the second part (three repetitions, two or more normal). The participants swallow 60 mL of water and observe completeness, the time required to complete swallowing, whether there is coughing during or after swallowing, whether there is wheezing during or after swallowing, and the function of the larynx after swallowing. The scale was scored on a scale of 5–12. The scale scores ranged from 18 to 46, and the higher the score, the worse the swallowing function (38). The high incidence of swallowing difficulties in mental illnesses such as AD has attracted attention. Currently, a research by Ye et al. (2024) has applied SSA to evaluate the swallowing function of patients with AD, demonstrating good reliability and validity (39). In this study, Cronbach’ s α of SSA was 0.914.

The Mini-Nutritional Assessment (MNA) scale was first proposed by Guigoz et al. in the 1990s to evaluate the nutritional status of the elderly. MNA includes 18 items and assesses four aspects: anthropometric measurements (BMI, weight loss, mid-arm and mid-calf circumferences); general assessment (related to lifestyle, medication, and mobility); dietary intake (related to number of meals, food and fluid intake, and autonomy of feeding) and subjective assessment (self-perception of health and nutrition). Scores less than 17 out of 30 are considered malnourished, scores between 17 and 23.5 indicate the risk of malnutrition, and scores of 24 or above are considered normal (40). In this study, Cronbach’ s α was 0.754.

#### 2.2.4 Care risk

The Nurses’ Global Assessment of Suicide Risk (NGASR) scale is an instrument which widely adopted by psychiatric nurses to assess suicide risk and make accurate, weighted clinical judgments (41). NGASR has been found to be quick and easy to administer, without need for preliminary training. NGASR consists of 15 items including recent negative life events, verbal expression of suicidal intent, feelings of despair, and history of suicide, each of which includes two answer choices of either “yes” or “no.” The five answers to items 1, 4, 7, 9, and 12 were scored as 3 points, the 10 answers to items 2, 3, 5, 6, 8, 10, 11, 13, 14, and 15 received 1 point, and all answers to items “no” were scored as 0 points. The total score was then obtained according to the rules of adding points. The maximum score of the scale is 25, with a total score of 0–5 representing low suicide risk, 6–8 representing medium suicide risk, 9–11 representing high suicide risk, and 12 representing very high suicide risk (42). In the study, Cronbach’ s α of NGASR was 0.760.

The Brøset Violence Checklist (BVC) was developed by Almvik et al. (1997) to assess the risk of violence in patients with inpatient psychiatric disorders. Each entry on the scale is given a score of 0 or 1 (none = 0; yes = 1). The scale measures the presence or absence of six behaviors: confusion, irritation, boisterousness, verbal threat, somatic threat, and destructive activity. The maximum total score was 6, with 0 representing the “lack of a behavior,” 1 being “its presence,” 6 being the “presence of a behavior”. The ratings ranged from 0 for low risk to 1–2 for moderate risk, indicating the need for preventative actions, to 3–6 for high risk, indicating the need for both preventive measures and a strategy to deal with violent aggression (43, 44). In the study, Cronbach’ s α was 0.739.

### 2.3 Data collection

This study adopted a questionnaire-survey method to collect data. Researchers who had completed consistency training distributed questionnaires in a tertiary psychiatric hospital. The filling time for questionnaire was approximately 25-30 minutes. General information and disease condition were accessed through hospital information system, and other information was collected in by patients with the assistance of researchers.

### 2.4 Data analysis

Statistical analysis was performed using the SPSS 25.0 statistical software. Measurement data was presented with mean ± sd, counting data was expressed using frequency and percentage. The difference between the two categories was performed by independent sample t-test, one-way ANOVA for three or more categories, and non-parametric K independent sample test for rank variable. Qualitative data is represented by composition ratios or rates, while intergroup comparisons are made using χ ^2^ inspection. And the level of significance was set at 0.05.

## 3 Results

### 3.1 General information

The 271 cases included 81 patients with schizophrenia, (26 males [32.1%], 55 females [67.9%]; 85 patients with depression (24 males [28.2%], 61 females [71.8%]), and 105 patients with AD (37 males [35.2%], 68 females [64.8%]). The female patients account for the largest proportion (67.9%), and those aged 60 to 69 are the most numerous (46.1%). The highest proportion of patients are those who are married (72.3%) (**Table 1**).

**Table 1:** General information.

	Schizophrenia	Depression	AD	Total
	n (%)/ Mean ± SD	n (%)/ Mean ± SD	n (%)/ Mean ± SD	n (%)/ Mean ± SD
Sex				
Male	26 (9.6%)	24 (8.9%)	37 (13.7%)	87 (32.1%)
Female	55 (20.3%)	61 (22.5%)	68 (25.1%)	184 (67.9%)
Age (years)				
60~69	54 (19.9%)	49 (18.1%)	22 (8.1%)	125 (46.1%)
70~79	25 (9.2%)	33 (12.2%)	41 (15.1%)	99 (36.5%)
≥80	2 (0.7%)	3 (1.1%)	42 (15.5%)	47 (17.3%)
Education background				
Illiteracy	5 (1.8%)	6 (2.2%)	6 (2.2%)	17 (6.3%)
Primary School	27 (10.0%)	26 (9.6%)	40 (14.8%)	93 (34.3%)
Middle School	29 (10.7%)	27 (10.0%)	36 (13.3%)	92 (33.9%)
High School	16 (5.9%)	14 (5.2%)	12 (4.4%)	42 (15.5%)
College and above	4 (1.5%)	12 (4.4%)	11 (4.1%)	27 (10.0%)
Marital Status				
Unmarried	6 (2.2%)	0 (0.0%)	1 (0.4%)	7 (2.6%)
Married	54 (19.9%)	64 (23.6%)	78 (28.8%)	196 (72.3%)
Remarried	1 (0.4%)	0 (0.0%)	1 (0.4%)	2 (0.7%)
Divorce	4 (1.5%)	3 (1.1%)	3 (1.1%)	10 (3.7%)
Widowed	16 (5.9%)	18 (6.6%)	22 (8.1%)	56 (20.7%)
Smoking				
No	75 (27.7%)	85 (31.4%)	88 (32.5%)	248 (91.5%)
Having Quitted Smoking	1 (0.4%)	0 (0.0%)	14 (5.2%)	15 (5.5%)
Yes	5 (1.8%)	0 (0.0%)	3 (1.1%)	8 (3.0%)
Drinking				
No	78 (28.8%)	84 (31.0%)	98 (36.2%)	260 (95.9%)
Yes	3 (1.1%)	1 (0.4%)	7 (2.6%)	11 (4.1%)
Type of Admission				
Referral from Emergency Centers	49 (18.1%)	25 (9.2%)	33 (12.2%)	107 (39.5%)
Outpatient Appointment	31 (11.4%)	55 (20.3%)	72 (26.6%)	158 (58.3%)
Referral from Other Hospitals	1 (0.4%)	5 (1.8%)	0 (0.0%)	6 (2.2%)
MECT				
No	81 (29.9%)	69 (25.5%)	105 (38.7%)	255 (94.1%)
Yes	0 (0.0%)	16 (5.9%)	0 (0.0%)	16 (5.9%)
Hospitalization Period (days)				
≤7	0 (0.0%)	1 (0.4%)	2 (0.7%)	3 (1.1%)
8~14	10 (3.7%)	11 (4.1%)	18 (6.6%)	39 (14.4%)
15~30	44 (16.2%)	49 (18.1%)	52 (19.2%)	145 (53.5%)
≥31	27 (10.0%)	24 (8.9%)	33 (12.2%)	84 (31.0%)

### 3.2 Mental status

The results showed that patients with depression had the highest GDS total score, followed by patients with AD, and patients with schizophrenia had the lowest score (*P* < 0.001). The total scores of GAD-7 and ITAQ in patients with depression were higher than those in patients with AD and schizophrenia (*P* < 0.001). The total score of MMSE in patients with schizophrenia and depression was higher than that in patients with AD (*P* < 0.001) (**Table 2**).

**Table 2:** Mental status.

	Schizophrenia	Depression	AD	Total	*F*	*P*	*Post hoc*	
	Mean ± SD	Mean ± SD	Mean ± SD	Mean ± SD				
GDS	9.95 ± 7.87	21.89 ± 7.43	14.25 ± 7.44	15.36 ± 8.92	54.479	<0.001**	2>3>1	2>3: LSD:7.65 ± 1.10, *P*<0.001** 3>1: LSD:4.30 ± 1.12, *P*<0.001** 2>1: LSD:11.94 ± 1.18, *P*<0.001**
GAD-7	5.41 ± 4.15	9.07 ± 5.04	5.16 ± 4.48	6.46 ± 4.88	20.274	<0.001**	2>1 = 3	2>1: LSD:3.66 ± 0.71, *P*<0.001** 2>3: LSD:3.91 ± 0.67, *P*<0.001**
BPRS	34.35 ± 5.72	33.51 ± 8.47	35.68 ± 8.90	34.60 ± 7.96	1.814	0.165	1 = 2 = 3	na
Anxiety/ Depression	6.27 ± 3.06	13.40 ± 4.67	6.74 ± 3.05	8.69 ± 4.83				
Withdrawal/ Retardation	5.35 ± 2.43	6.47 ± 3.41	10.65 ± 4.30	7.75 ± 4.24				
Thinking disorder	9.80 ± 3.18	5.66 ± 2.19	7.34 ± 3.32	7.55 ± 3.38				
Activation	4.14 ± 1.77	4.00 ± 1.63	4.18 ± 1.86	4.11 ± 1.76				
Suspicion	8.79 ± 3.33	3.98 ± 2.25	6.76 ± 3.39	6.49 ± 3.59				
ITAQ	4.01 ± 5.52	8.79 ± 6.10	3.29 ± 3.23	5.23 ± 5.52	32.120	<0.001**	2>1 = 3	2>1: LSD:4.78 ± 0.77, *P*<0.001** 2>3: LSD:5.50 ± 0.73, *P*<0.001**
MMSE	22.11 ± 6.56	22.39 ± 6.18	9.15 ± 5.77	17.18 ± 8.85	146.382	<0.001**	1 = 2>3	1>3: LSD:12.96 ± 0.91, *P*<0.001** 2>3: LSD:13.24 ± 0.90, *P*<0.001**
Orientation	7.69 ± 2.75	7.92 ± 2.47	2.28 ± 2.36	5.66 ± 3.69				
Registration	2.69 ± 0.65	2.66 ± 0.73	1.58 ± 1.22	2.25 ± 1.07				
Attention and Calculation	2.86 ± 1.61	3.08 ± 0.65	0.83 ± 1.14	2.14 ± 1.80				
Recall	1.74 ± 1.00	1.64 ± 0.95	0.32 ± 0.67	1.16 ± 1.09				
Object Naming	1.88 ± 0.40	1.85 ± 0.39	1.54 ± 0.72	1.74 ± 0.57				
Verbal Rehearsal	0.99 ± 0.11	0.95 ± 0.21	0.70 ± 0.46	0.86 ± 0.34				
Reading Comprehension	0.93 ± 0.26	0.87 ± 0.34	0.47 ± 0.50	0.73 ± 0.44				
Verbal Comprehension	2.35 ± 0.96	2.51 ± 0.93	1.06 ± 1.60	1.90 ± 1.23				
Verbal Expression	0.47 ± 0.50	0.44 ± 0.50	0.23 ± 0.42	0.37 ± 0.48				
Graphic Drawing	0.52 ± 0.50	0.48 ± 0.50	0.15 ± 0.36	0.37 ± 0.48				

“1”=Schizophrenia; “2”=Depression; “3”=AD.

GDS, Geriatric Depression Scale; GAD-7, Generalized Anxiety Disorder 7-Item Scale; BPRS, Brief Psychiatric Rating Scale; ITAQ, Insight and Treatment Attitudes Questionnaire; MMSE, Mini-Mental State Examination.

**P<0.001.

### 3.3 Somatic diseases

The results showed that approximately 60% of respondents had been diagnosed with at least two types of somatic diseases. The three most frequent diseases were from cardiovascular (54.6%), endocrine (36.2%), and nervous (35.1%) systems. The incidence of circulatory system diseases in patients with depression and AD was higher than that in patients with schizophrenia (*P* < 0.05). The incidence of respiratory system diseases in patients with AD was highest, followed by patients with schizophrenia, and patients with depression had the lowest incidence (*P* < 0.05). The incidence of nervous system diseases in patients with AD was highest, followed by patients with depression, and patients with schizophrenia had the lowest incidence (*P* < 0.05) (**Table 3**).

**Table 3:** Somatic diseases.

	Schizophrenia	Depression	AD	Total	*χ^2^*	*P*	*Post hoc*
	n (%)	n (%)	n (%)	n (%)			
Numbers of Somatic Diseases							
0	16(5.9%)	8(3.0%)	12(4.4%)	36(13.3%)	21.512	0.089	na
1	21(7.7%)	28(10.3%)	20(7.4%)	69(25.5%)			
2	22(8.1%)	30(11.1%)	27(10.0%)	79(29.2%)			
3	14(5.2%)	14(5.2%)	24(8.9%)	52(19.2%)			
4	5(1.8%)	4(1.5%)	13(4.8%)	22(8.1%)			
5	3(1.1%)	1(0.4%)	7(2.6%)	11(4.1%)			
6	0(0.0%)	0(0.0%)	1(0.4%)	1(0.4%)			
7	0(0.0%)	0(0.0%)	1(0.4%)	1(0.4%)			
Circulatory System							
No	46(17.0%)	35(12.9%)	42(15.5%)	123(45.4%)	6.086	0.048*	2=3>1
Yes	35(12.9%)	50(18.5%)	63(23.2%)	148(54.6%)			
Digestive System							
No	69(25.5%)	72(26.6%)	93(34.3%)	234(86.3%)	0.728	0.695	na
Yes	12(4.4%)	13(4.8%)	12(4.4%)	37(13.7%)			
Respiratory System							
No	58(21.4%)	68(25.1%)	63(23.2%)	189(69.7%)	9.094	0.011*	3>1>2
Yes	23(8.5%)	17(6.3%)	42(15.5%)	82(30.3%)			
Urinary System							
No	75(27.7%)	81(29.9%)	96(35.4%)	252(93.0%)	1.104	0.576	na
Yes	6(2.2%)	4(1.5%)	9(3.3%)	19(7.0%)			
Motor System							
No	72(26.6%)	74(27.3%)	96(35.4%)	242(89.3%)	0.959	0.619	na
Yes	9(3.3%)	11(4.1%)	9(3.3%)	29(10.7%)			
Endocrine System							
No	52(19.2%)	62(22.9%)	59(21.8%)	173(63.8%)	5.716	0.057	na
Yes	29(10.7%)	23(8.5%)	46(17.0%)	98(36.2%)			
Immune System							
No	76(28.0%)	82(30.3%)	98(36.2%)	256(94.5%)	0.974	0.614	na
Yes	5(1.8%)	3(1.1%)	7(2.6%)	15(5.5%)			
Nervous System							
No	61(22.5%)	59(21.8%)	56(20.7%)	176(64.9%)	10.785	0.005*	3>2>1
Yes	20(7.4%)	26(9.6%)	49(18.1%)	95(35.1%)			
Reproductive System							
No	78(28.8%)	81(29.9%)	96(35.4%)	255(94.1%)	2.270	0.321	na
Yes	3(1.1%)	4(1.5%)	9(3.3%)	16(5.9%)			

“1”=Schizophrenia; “2”=Depression; “3”=AD.

*P<0.05.

### 3.4 Social and daily living function

The FAQ, SSRS, Barthel ADL Index, water-swallowing test, SSA, and MNA scale were used to evaluate the patients’ social and daily activities. The outcomes demonstrated that the total scores of FAQ and SSA in patients with AD were higher than those in patients with schizophrenia and depression (*P* < 0.001), while patients with depression had statistically lower SSRS scores than patients with schizophrenia and patients with AD (*P* < 0.05). Furthermore, patients with AD had lower Barthel ADL Index scores and water-swallowing test (*P* < 0.001). MNA scores of patients with schizophrenia were higher than those of patients with depression and AD, with statistical significance (*P* < 0.05) (**Table 4**).

**Table 4:** Social and daily living function.

	Schizophrenia	Depression	AD	Total	*F*	*P*	*Post hoc*	
	n (%)/ Mean ± SD	n (%)/ Mean ± SD	n (%)/ Mean ± SD	n (%)/ Mean ± SD				
FAQ	7.73 ± 9.36	7.44 ± 7.83	23.02 ± 8.63	13.56 ± 11.42	103.255	<0.001**	3>1 = 2	3>1: LSD:15.29 ± 1.27, *P*<0.001** 3>2: LSD:15.58 ± 1.26, *P*<0.001**
SSRS	27.60 ± 5.14	23.02 ± 8.63	29.07 ± 5.98	29.34 ± 5.97	8.801	<0.001**	1 = 3>2	1>2: LSD:3.74 ± 0.90, *P*<0.001** 3>2: LSD:2.28 ± 0.85, *P=*0.008*
Objective Support	8.40 ± 2.13	9.40 ± 2.54	8.03 ± 1.98	8.57 ± 2.28				
Subjective Support	15.30 ± 3.30	17.31 ± 3.66	15.98 ± 3.28	16.19 ± 3.49				
Utilization of Support	3.91 ± 1.57	4.64 ± 2.01	5.06 ± 2.37	4.58 ± 2.09				
Barthel ADL Index	87.16 ± 17.21	81.59 ± 19.70	55.86 ± 28.69	73.28 ± 26.89	50.366	<0.001**	1 = 2>3	1>3: LSD:31.30 ± 3.40, *P*<0.001** 2>3: LSD:25.73 ± 3.36, *P*<0.001**
Food Intake	8.95 ± 2.33	8.59 ± 2.85	5.43 ± 3.54	7.47 ± 3.41				
Dressing	8.46 ± 2.45	7.71 ± 3.23	4.24 ± 3.45	6.59 ± 3.64				
Going to Lavatory	8.27 ± 2.64	7.88 ± 3.12	4.43 ± 3.56	6.66 ± 3.63				
Shower	3.27 ± 2.39	2.29 ± 2.51	0.71 ± 1.76	1.97 ± 2.45				
Decoration	3.83 ± 2.13	3.59 ± 2.27	1.38 ± 2.25	2.80 ± 2.49				
Controlling of Excrement	9.81 ± 0.95	9.53 ± 1.66	7.57 ± 3.68	8.86 ± 2.72				
Control of Urine	9.63 ± 1.54	9.41 ± 1.96	7.33 ± 3.86	8.67 ± 2.96				
Bed-Chair Transfer	13.83 ± 2.41	13.65 ± 2.49	10.43 ± 5.24	12.45 ± 4.10				
Ground Walking	13.70 ± 2.71	13.12 ± 3.18	10.43 ± 5.38	12.25 ± 4.32				
Stairs Walking	7.72 ± 3.07	6.53 ± 3.28	4.43 ± 3.75	6.07 ± 3.67				
Water Swallow Test	-		-		53.323	<0.001**	1 = 2>3	1>3: LSD:0.99 ± 0.11, *P*<0.001** 2>3: LSD:0.91 ± 0.11, *P*<0.001**
I	79(29.2%)	79(29.2%)	37(13.7%)	195(72.0%)				
II	1(0.4%)	4(1.5%)	45(16.6%)	50(18.5%)				
III	1(0.4%)	1(0.4%)	10(3.7%)	12(4.4%)				
IV	0(0.0%)	0(0.0%)	9(3.3%)	9(3.3%)				
V	0(0.0%)	1(0.4%)	4(1.5%)	5(1.8%)				
SSA	18.20 ± 1.07	18.06 ± 0.32	21.80 ± 4.96	19.55 ± 3.62	43.758	<0.001**	3>1 = 2	3>1: LSD:3.60 ± 0.47, *P*<0.001** 3>2: LSD:3.47 ± 0.46, *P*<0.001**
Clinical Assessment	7.05 ± 0.35	7.00 ± 0.00	8.29 ± 2.00	7.51 ± 1.40				
Stage 1	6.05 ± 0.31	6.01 ± 0.11	7.12 ± 1.61	6.45 ± 1.45				
Stage 2	5.10 ± 0.51	5.05 ± 0.26	6.39 ± 1.85	5.58 ± 1.36				
MNA	8.80 ± 2.15	7.49 ± 2.95	6.97 ± 2.45	7.68 ± 2.64	12.237	<0.001**	1>2 = 3	1>2: LSD:1.31 ± 0.39, *P=*0.001* 1>3: LSD:1.83 ± 0.38, *P*<0.001**

“1”=Schizophrenia; “2”=Depression; “3”=AD.

FAQ, The Functional Activities Questionnaire; SSRS, Social Support Rating Scale; ADL, Activities of Daily Living.

SSA, Standardized Swallowing Assessment; MNA, Mini-Nutritional Assessment.

*P<0.05; **P<0.001.

### 3.5 Nursing risk

The NGASR and BVC were used to assess the patients’ risk of suicide. The results revealed that the NGASR scores of patients with depression were higher than those of patients with schizophrenia and AD, which was statistically significant (*P* < 0.001). Patients with AD had the highest BVC total score, followed by that of patients with schizophrenia and patients with depression had lowest score, and the difference was statistically significant (*P* < 0.05) (**Table 5**).

**Table 5:** Nursing risk.

	Schizophrenia	Depression	AD	Total	*F*	*P*	*Post hoc*	
	Mean ± SD	Mean ± SD	Mean ± SD	Mean ± SD				
NGASR	3.60 ± 3.06	7.18 ± 4.32	2.70 ± 2.47	4.38 ± 3.83	45.827	<0.001**	2>1 = 3	2>1: LSD:3.57 ± 0.52, *P*<0.001** 2>3: LSD:4.47 ± 0.48, *P*<0.001**
Despair	0.42 ± 1.04	1.64 ± 1.50	0.11 ± 0.58	0.68 ± 1.25				
Negative Life Events	0.04 ± 0.19	0.16 ± 0.37	0.04 ± 0.19	0.08 ± 0.27				
Delusion & Hallucination	0.73 ± 0.45	0.19 ± 0.48	0.33 ± 0.53	0.41 ± 0.54				
Depressed	0.48 ± 1.11	2.27 ± 1.29	0.95 ± 1.40	1.23 ± 1.47				
Social Functioning Declines	0.32 ± 0.54	0.40 ± 0.49	0.17 ± 0.38	0.29 ± 0.48				
Suicidal Intention	0.11 ± 0.32	0.54 ± 0.63	0.04 ± 0.19	0.22 ± 0.46				
Suicidal Plan	0.11 ± 0.57	0.51 ± 1.12	0.01 ± 0.10	0.20 ± 0.73				
Family History of Suicide	0.01 ± 0.11	0.05 ± 0.34	0.01 ± 0.10	0.02 ± 0.21				
Loss of Significant Relationship	0.22 ± 0.79	0.39 ± 1.01	0.46 ± 1.08	0.37 ± 0.98				
History of Psychiatry	0.67 ± 0.47	0.35 ± 0.48	0.26 ± 0.50	0.41 ± 0.52				
Widowed	0.16 ± 0.37	0.14 ± 0.44	0.21 ± 0.41	0.17 ± 0.41				
Attempted Suicide	0.30 ± 0.90	0.53 ± 1.15	0.07 ± 0.42	0.28 ± 0.87				
Low Socioeconomic Status	0.01 ± 0.11	0.00 ± 0.00	0.01 ± 0.10	0.01 ± 0.09				
Substance Abuse	0.02 ± 0.16	0.01 ± 0.11	0.02 ± 0.14	0.02 ± 0.14				
Terminal Illness	0.00 ± 0.00	0.00 ± 0.00	0.02 ± 0.14	0.01 ± 0.09				
BVC	1.56 ± 1.80	0.27 ± 0.76	2.27 ± 1.50	1.43 ± 1.64	46.969	<0.001**	3>1>2	3>1: LSD:0.71 ± 0.21, *P=*0.001* 1>2: LSD:1.28 ± 0.22, *P*<0.001** 3>2: LSD:2.00 ± 0.21, *P*<0.001**
Confusion	0.16 ± 0.37	0.09 ± 0.29	0.89 ± 0.32	0.42 ± 0.50				
Irritated	0.42 ± 0.50	0.07 ± 0.26	0.57 ± .50	0.37 ± 0.48				
Noisy	0.37 ± 0.49	0.04 ± 0.19	0.40 ± 0.49	0.28 ± 0.45				
Physical Threat	0.16 ± 0.37	0.05 ± 0.21	0.12 ± 0.33	0.11 ± 0.31				
Verbal Threat	0.21 ± 0.41	0.01 ± 0.11	0.13 ± 0.34	0.12 ± 0.32				
Destruction	0.23 ± 0.43	0.01 ± 0.11	0.15 ± 0.36	0.13 ± 0.34				

“1”=Schizophrenia; “2”=Depression; “3”=AD.

NGASR, The Nurses’ Global Assessment of Suicide Risk; BVC, The Brøset Violence Checklist.

*P<0.05; **P<0.001.

## 4 Discussion

Understanding the clinical characteristics of mental disorders among older adults can help medical professionals to rationally allocate medical resources as well as to develop nursing care plans. Our finding suggested that depression, schizophrenia, and AD were three most prevalent mental disorders among older adults. Previous studies by Huang et al. (2019) reported that among adults aged above 65 years, the weighted lifetime prevalence of AD was more than 5%, followed by depression and schizophrenia (45). In addition to mental disorders, a number of older adults suffered from somatic discomfort, for instance chronic pain, weakness, and malnutrition (46). Moreover, older people were found to be more prone to experience adverse life events including retirement, widowhood, infirmity, and loss of independence. Such events can result in experiencing psychological emotions like hopelessness and loneliness. Owing to a variety of biological, psychological, and social factors, older adults had a lower capacity for life adaptation, which might have a negative psychological impact and even trigger mental disorders.

Almost half of respondents were diagnosed with at least three somatic diseases, and circulatory diseases were the most threatening. Physical condition should be a vital factor influencing mental health among older adults. The decline of physical health was found to result in poor mental well beings. Therefore, applying interventions to relieving somatic diseases is significant to improve the mental health among older adults. Our findings highlighted that the majority of aged psychiatric patients (55.7%) had been affected by circulatory diseases. Ungvari et al. (2021) noted that the health conditions of older adults diagnosed with hypertension were more likely to be complicated with cognitive impairments (47). Therefore, in regard to mental health care for older adults, despite the chief complaint of mental illnesses, attention should be paid to patients’ physical condition as well, particularly to various circulatory diseases (48). In addition, we found that the proportion of respiratory system diseases and nervous system diseases among patients with AD was higher compared to schizophrenia and depression. A study by Agnieszka et al. (2024) showed that patients with AD experienced respiratory disturbances (49), which was consistent with our results. Respiratory system disorders are a frequent cause of mortality in patients with AD, potentially attributable to diminished respiratory muscle strength. AD is a chronic, progressive neurodegenerative disorder characterized by neurodegenerative changes in the brain. These changes could adversely affect lung function, contributing to respiratory system diseases. Conversely, impaired lung function leads to hypoxia, which might exacerbate neurodegenerative processes and accelerate the progression of dementia. Therefore timely detection and treatment of respiratory infections is important. In addition, nursing staff should regularly assess cognitive function and develop targeted cognitive training plans based to delay the decline of cognitive function, such as memory training, attention training (49).

Adequate care requirements should be offered to individuals who have been diagnosed with varied mental diseases. The results of this study demonstrated that the MMSE score of schizophrenic patients was (22.11 ± 6.56), indicating that they had cognitive impairment. Our result was in consistent with the study by Goonathilake et al. (2022) (50). Patients with schizophrenia were typically distressed by hallucinations and delusions, such psychotic symptoms would impair individuals’ ability to perceive reality objectively and lead to cognitive impairment. In relation to schizophrenia, neurodevelopmental theories proposed, abnormal brain development could impede the acquisition of cognitive abilities throughout the ontogenetic process, thus neurodevelopmental abnormalities might serve as one of critical reasons of the cognitive deficits in individuals with schizophrenia (51, 52). Furthermore, patients with schizophrenia experience worse decline in cognitive abilities in later life, this might due to the adverse effects on cerebrovascular function caused by unhealthy lifestyle, obesity, or hyperglycemia (53, 54). Noticeably, the study by Zhu et al. (2022) reported that amisulpride augmentation therapy improved cognitive performance and psychopathology in clozapine-resistant treatment-refractory schizophrenia, however further research evaluating the effectiveness of amisulpride augmentation therapy in older adults are needed (55). Besides, this study demonstrated that elderly patients with depression, and AD exhibited significant psychiatric symptoms as well. Previous research had identified substantial symptom overlap among various mental disorders. For instance, cognitive impairments in domains such as attention, social cognition, and working memory were common in conditions like schizophrenia and severe depression. This overlap in symptoms might be partially attributed to genetic connections between these mental illnesses (56). Compared with patients with schizophrenia and depression, patients with AD had lower Barthel ADL index scores and worse swallowing function, based on study’s findings. As opposed to the other two sorts of patients, they scored higher on the FAQ and SSA. This was in accordance with earlier studies that have demonstrated a progressive decline in patients with AD’ capacity to perform daily tasks or even live independently. Due to mental disorder, the diminished capacity might be ascribed to impaired cognitive and judgmental abilities among patients with AD. Furthermore, several previous cohort studies had demonstrated a correlation between the intensity of symptoms experienced by patients with AD and the degree of decrease in their capacity to carry out activities of daily living (57). Dysphagia is common in dementia patients. In individuals with dementia, the voluntary phases of swallowing, particularly the oral preparation and oral phases, are impaired due to cognitive deficits that hinder voluntary motor control (58, 59). To realize the autonomy and independence of patients with AD as soon as possible, adequate nursing measures should be adopted at different stages of the disease (60). As a result of the dysfunction brought on by the condition, people with AD have a significant requirement for food intake and personal hygiene since they have a considerably diminished capacity to take care of themselves. The results of this study, which showed that patients with depression had a higher NGASR score than patients with schizophrenia or AD, our finding was consistent with the researches indicating that hospitalized patients with depression were at greater risk of suicide than patients with other mental disorders (61). A study included psychological autopsy found that more than half of suicide victims exhibited characteristics of depression at some point in their lives (62). This could be explained by the fact that individuals with depression frequently displayed negative attitudes, pessimism, and suicidal thoughts and actions. Thus, the main clinical focus of psychiatric caregivers should be prompt assessment of patients with depression who were at high risk of suicide and appropriate care interventions to lower the risk. Hence, extensive nursing care should be provided to elderly patients who are depressed. Nurses should closely monitor patient’s abnormal bahavior and react promptly to an emergency and avoid suicide. In addition, compared to the other two types of disorders, patients with AD had a higher risk of violence. The aggressive and destructive behavior of elderly patients with AD might cause considerable pressure on caregivers. This might be related to the severe decline in cognitive abilities of patients with AD as the disease progresses, which affects their ability to think clearly and communicate effectively, ultimately leading to the occurrence of violence. Effective communication and moderation techniques could help prevent and mitigate aggressive incidents. Besides, providing support and education for nursing staff is crucial. The goal of intervention is to create a safe and supportive environment for patients, and to adopt a compassionate and multi-faceted approach to managing aggressive behavior (63).

## 5 Limitations

Limitations of this study should be noted. This study is a retrospective study of elderly patients admitted to the psychiatric department continuously. Participants were recruited from Brain Hospital Affiliated to Guangzhou Medical University, this might have selection bias and recall bias. In this study, the symptom severity of the same diseases varies greatly from the samples, the sample collection time span was large, and the symptom manifestations may be different in different seasons, which might affect its objectivity. In future studies, sample collection should be done from the same source and in a concentrated time to improve sample homogeneity.

## 6 Conclusions

In conclusion, a great proportion of elderly individuals with mental illness might experienced irregularities in their thinking, cognition, emotions, and behavior. The manifestations of various types of mental illness had diverse clinical characteristics, physical sickness frequently coexists with mental illness in elderly individuals. Treatment of various types of mental disorders, such as employing various nursing interventions, is crucial for aged adults with mental illnesses.

## 7 Implication for nursing

The results of this study help nursing staff understand the clinical characteristics of hospitalized elderly patients with mental disorders, provide evidence for the future prevention and treatment of elderly schizophrenia, emotional disorders, and AD, and also provide ideas for the development of effective intervention measures. The adoption of various nursing interventions is crucial for all types of mental illness.

## Data Availability

The raw data supporting the conclusions of this article will be made available upon reasonable request to the corresponding author.
